# Severe maternal hardships are associated with food insecurity among low-income/lower-income women during pregnancy: results from the 2012–2014 California maternal infant health assessment

**DOI:** 10.1186/s12884-022-04464-x

**Published:** 2022-02-19

**Authors:** Barbara A. Laraia, Ryan Gamba, Carina Saraiva, Melanie S. Dove, Kristen Marchi, Paula Braveman

**Affiliations:** 1 Division of Community Health Sciences, Public Health Nutrition Program, 2121 Berkeley Way West #6126, Berkeley, CA USA; 2 School of Public Health, University of California, Berkeley, CA USA; 3 Department of Health Sciences, California State East Bay, 25800 Carlos Bee Boulevard, Hayward, CA 94542 USA; 4 Maternal, Child and Adolescent Health Program, California Department of Public Health, Sacramento, CA 95899 USA; 5 Department of Public Health Sciences, University of California, Davis, 4150 V Street, Suite 2400, Sacramento, CA 95817 USA; 6 Center for Health Equity, Department of Family and Community Medicine, University of California, San Francisco, 3333 California Street, Suite 365, Box 0943, San Francisco, CA 94143 USA; 7 Center for Health Equity, Department of Family and Community Medicine, University of California, San Francisco, 3333 California Street, Suite 365, Box 0943, San Francisco, CA 94143 USA

**Keywords:** Food Insecurity, Pregnancy, Maternal Hardship

## Abstract

**Background:**

Assess the associations between ten severe maternal hardships and food insecurity experienced during pregnancy.

**Methods:**

Data on 14,274 low-income/lower-income women (below 400% of the income to federal poverty guideline ratio) from the statewide-representative 2010–2012 California Maternal and Infant Health Assessment were used to estimate food security status prevalence. Prevalence of severe maternal hardships by food security status was estimated. Multinomial logistic regression was used to assess the associations between severe maternal hardship and food security status, adjusting for sociodemographic characteristics.

**Results:**

Food insecurity was common among low- and lower-income pregnant women in California; 23.4% food insecure and an additional 11.5% marginally secure. In adjusted analysis, nine of ten hardships were associated with food security status. Only the respondent or someone close to the respondent having a problem with alcohol or drugs was not associated with food security status after adjusting for socioeconomic factors. Husband/partner losing a job, depressive symptoms, not having practical support and intimate partner violence were consistently associated with marginal, low and very low food security status. Each additional severe maternal hardship a woman experienced during pregnancy was associated with a 36% greater risk of reporting marginal food security (Relative Risk Ratio 1.36, 95% CI: 1.27, 1.47), 54% for low food security (Relative Risk Ratio 1.54, 95% CI: 1.44, 1.64), and 99% for very low food security (Relative Risk Ratio 1.99, 95% CI: 1.83, 2.15).

**Conclusions:**

Food security status was strongly linked with several maternal hardships that could jeopardize maternal and/or infant health.

Services—including prenatal care and nutritional assistance—for a large proportion of pregnant women should address a wide range of serious unmet social needs including food insecurity.

## Background

Food insecurity is a multidimensional health risk that includes anxiety about, a lack of material resources for, and poor access to nutritious foods that can have implications across the lifecourse. Prior to and during the Great Recession, 1999–2010, prevalence estimates of food insecurity among pregnant American women were scarce and range from to 15% to 18.3% [1–4]. During pregnancy, food insecurity is associated with maternal depression, anxiety, perceived stress, disordered eating [3], and may increase risk for greater gestational weight gain and gestational diabetes [5, 6]. For the growing fetus, food insecurity has been associated with low birth weight [7], birth defects (i.e., cleft palate, d-transposition of the great arteries, tetralogy of Fallot, spina bifida, and anencephaly) [8], diabetes and coronary heart disease [9]. The mechanism of these associations has been hypothesized to be stress caused by a lack of food in the presence of nutrient deficiencies. These associations have established food insecurity as a primary risk exposure for the new Association of Maternal and Child Health Programs sponsored Life Course Metrics Project aimed to identify and establish standardized metrics for the life course approach [10].

Taking a life-course approach, maternal hardships experienced during pregnancy may have short- and long-term health consequences [10]. Severe maternal hardships are associated with elevated levels of stress, poor eating behaviors, and weight gain [11, 12] that may affect the mother’s health later in her life. For the infant, these influence birth outcomes [13], and children exposed in utero to severe maternal stress have been shown to have poor stress management later in life [14, 15] and to have developed insulin resistance [16]. The California Maternal and Infant Health Assessment [17], a statewide-representative survey of women who recently had a live birth, revealed that during 2002–2006 not only did 18% of women report food insecurity, but, in addition, 18% reported having more bills than they could pay, 11% had spouses or partners who lost a job, 9% had their own involuntary job loss, 7% became separated or divorced during pregnancy, 3.3% experienced domestic violence, and 3.2% experienced the incarceration of themselves or their spouse/partner during pregnancy [4].

The Centers for Disease Control and Prevention (CDC)’s Pregnancy Risk Assessment Monitoring System (PRAMS) retrospectively assesses 13 maternal hardships as part of the core questionnaire [18, 19] but the optional one-item food sufficiency question has been asked in only six states and state prevalences have not been reported. Food insecurity is a common economic hardship faced by pregnant women but is often not evaluated in clinical settings. Few real-time surveillance systems directly assess the prevalence of food insecurity, the degree of its severity, or attendant risks during pregnancy.

Food insecurity is a relatively common hardship that may co-exist with a number of additional severe maternal hardships such as other financial hardships, adverse life events, and emotional stressors [4, 20]. Despite this, the association between severe maternal hardships and food security status during pregnancy has not, to our knowledge, been explored using a population-based sample. It is important to document the prevalence and severity of food insecurity and accompanying maternal hardship to inform policies and identify the resources needed by programs serving pregnant women who experience food insecurity. The objective of this paper was to assess the extent to which severe maternal hardships were associated with food insecurity during pregnancy among a representative sample of low- and lower-income women who had live births in California post the Great Recession in 2010–2012.

## Methods

MIHA [17] is an annual, statewide cross-sectional survey of a representative sample of California women who recently had a live birth, excluding women under the age of 15 years, non-residents, and women with multiple births greater than three. MIHA is conducted by the California Department of Public Health using federal Title V funds. The data used for this project were authorized under an administrative contract with the California Department of Public Health and are currently not available to researchers outside of the California Department of Public Health.

MIHA uses random sampling, stratified on county/region of residence, African- American race, and, during the study period of interest, enrollment in the Women, Infant and Child Supplemental Nutrition program. Survey data was collected from 20,480 women who recently gave a live birth during 2010–2012. MIHA maintained an annual response rate of approximately 70% over the three years. The MIHA data were weighted to represent all California- resident women 15 years of age and older in California with a singleton, twin or triplet live birth during each survey year. We excluded 3,108 sampled women with household incomes above 400% of the Federal Poverty Guidelines (FPG) (e.g., $74,000 for a family of three in 2011) [21] slightly higher than the median household income for California of $70,400 [22] because few women (< 0.6%) with household incomes > 400% FPG experienced food insecurity. In addition, women with missing household income information were initially excluded, although these values were later imputed for sensitivity analyses (*n* = 1,479), had missing food security information (*n* = 112), or were in a stratum with a single sampling unit (*n* = 2). These 15,779 observations were used to estimate the overall prevalence of food security status. Women were excluded from additional analyses if they reported a racial/ethnic group other than White, Black, Latina, or Asian/Pacific Islander (*n* = 899) or had incomplete maternal hardship information (*n* = 606). The final analytic sample consisted of 14,274 women.

### Dependent variable—food insecurity

During the study period, the MIHA survey incorporated the validated 6-item food security scale developed by the United States Department of Agriculture [23], slightly modified to ask about the woman’s experience with food security during her most recent pregnancy rather than the past 12 months (Table 1). [4] Not responding affirmatively to any of the six questions indicated the woman was food secure during pregnancy. One affirmative response indicated marginal food security. [24] More than one affirmative response indicated food insecurity, further defined as low food security (2–4 affirmative responses) or very low food security (5–6 affirmative responses).

**Table 1:** Questions used to assess Food Security Status during pregnancy in the Maternal Infant Health Assessment Survey

Food Security Questions	Affirmative response
1.“The food that I bought just didn't last, and I didn't have money to get more." During your most recent pregnancy, was that often, sometimes, or never true for you?	Often or sometimes
2."I couldn't afford to eat balanced meals." During your most recent pregnancy, was that often, sometimes, or never true for you?	Often or sometimes
3.During your pregnancy, did you ever cut the size of your meals or skip meals because there wasn't enough money for food?	Yes
4.During your pregnancy, did you ever eat less than you felt you should because there wasn't enough money to buy food?	Yes
5.How often did this [cut size or skip meals] happen?	Three or more times per month
6.During your pregnancy, were you ever hungry but didn't eat because you couldn't afford enough food?	Yes

### Covariates

Covariates included year of survey (2010, 2011, 2012), age (15–19, 20–24, 25–29, 30–34, ≥ 35 years), educational attainment (less than high school diploma, high school diploma or GED, some college, college graduate or more), primary language spoken at home (English, Spanish, other), number of people living on the household income (≤ 2, 3–4, ≥ 5), household income as a percent of federal poverty guidelines (0 -100%, 101—200%, 201—300%, 301—400% FPG), health insurance status during pregnancy (Medi-Cal [California’s Medicaid program], private, uninsured, other), and marital status (married, living with someone, single). For race/ethnicity, there were five mutually exclusive groups (White, Latina US-born, Latina foreign-born, Black, Asian/Pacific Islander); Latinas were the only group with enough US-born and foreign-born women to create two groups based on nativity. There were too few women of American Indian or “other” race to include in these analyses.

Apart from food insecurity, MIHA assesses ten additional severe maternal hardships including financial hardships, adverse life events, and emotional stressors. Participants responded to the statement: “Here are a few things that might happen to some women during their pregnancies. Please tell us if any of these things happened to you during your most recent pregnancy.” All response options were yes/no. *Financial hardships* during pregnancy included: the respondent losing a job or the respondent’s husband/partner losing a job, or the respondent reporting being homeless or not having a regular place to sleep. *Adverse life events* during pregnancy included: the respondent becoming separated or divorced, someone close to the respondent having an alcohol or drug problem, someone close to the respondent going to jail, or the respondent experiencing intimate partner violence. *Emotional Stressors* during pregnancy included: not having someone she could turn to for emotional support (e.g., someone to listen to or comfort her when needed), not having someone she could turn to if she needed practical help (e.g., like getting a ride somewhere or help with shopping or cooking a meal), and experiencing depressive symptoms. We also created a continuous variable of the total number of maternal hardships (0–10) each woman experienced during her pregnancy.

### Analysis

Prevalence estimates and confidence intervals of maternal characteristics and food security status were estimated using survey-weighted tabulations. Chi squared test was used to assess the bivariate relationship between food security status and each maternal hardship. Multinomial logistic regression was implemented to estimate the relative risk ratios for the ten severe maternal hardships in relation to being marginally food secure, low food secure, or very low food secure compared to food secure. The model accounted for year of survey, maternal race/ethnicity, age, education, language spoken at home, number of people in the household, income as the percent of the poverty guidelines, insurance coverage, and marital status.

Missing income was imputed using ordered logistic regressions across five imputations and sensitivity analyses were conducted with the imputed datasets to determine if our findings were robust after accounting for the missing values. All socioeconomic/demographic, hardship, and food security data were included in the imputation model. The imputed datasets contained an additional 875 women. All analyses were conducted using Stata (version 12.0, StataCorp, College Station, TX).

The research for this study was conducted with approval from two Institutional Review Boards: the California Department of Health and Human Services, and the University of California, San Francisco. All methods were performed in accordance with the relevant guidelines and regulations of these institutions. The elements of informed consent were provided to potential participants in writing via the mail, or over the phone. Per common practice, consent was assumed either by voluntary return of a mail survey or by willingness to continue the interview after being asked.

## Results

**Fig. 1:**
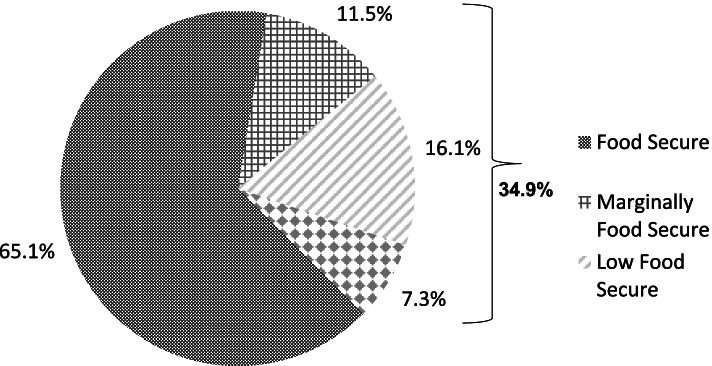
Prevalence of food insecurity among pregnant women in MIHA with household incomes ≤ 400% of the Federal Poverty Guidelines (*n* = 15,779)

Among women with household incomes ≤ 400% FPG, 65.1% reported being fully food secure during pregnancy and 34.9% reported some level of food insecurity (Fig. 1). The prevalence of food insecurity was 23.4%; with 16.1% experiencing low food security and 7.3% experiencing very low food security. An additional 11.5% reported marginal food security.

Table 2 provides a description of the analytic sample of pregnant women with household incomes ≤ 400% FPG who participated in MIHA 2010 – 2012 and who had complete information on the variables that were studied. A higher percent of women reported food insecurity who: were Latina or Black compared to white, younger compared to older age, had a lower educational level compared to college graduate; primarily spoke Spanish compare to English at home; had five or more people compared to four or less dependent on the household income; had income below compared to above the federal poverty level; were either uninsured or lacked private insurance compared to those with private insurance; or were not married compared to married.

**Table 2:** Sample characteristics and severe maternal hardship by food security status among postpartum women with household incomes ≤ 400% of the Federal Poverty Guidelines in California, MIHA 2010 – 2012 (*n* = 14,274)

		**Distribution in Total Sample**	**Food Secure**	**Marginally Food Secure**	**Low Food Secure**	**Very Low Food Secure**
	**n**	**Col.%, (95% CI)**	**Row%, (95% CI)**	**Row%, (95% CI)**	**Row%, (95% CI)**	**Row%, (95% CI)**
**Total**	14,273	100	66.2 (64.7, 67.6)	11.4 (10.4, 12.5)	15.8 (14.7, 17.0)	6.6 (5.9, 7.5)
**Year**						
2010	4835	34.2 (33.3, 35.1)	65.0 (62.4, 67.5)	11.9 (10.3, 13.7)	16.1 (14.3, 18.2)	7.0 (5.7, 8.5)
2011	4815	33.2 (32.3, 34.2)	65.7 (63.1, 68.2)	11.6 (9.8, 13.6)	16.0 (14.2, 17.9)	6.8 (5.6, 8.1)
2012	4623	32.6 (31.6, 33.6)	67.9 (65.4, 70.4)	10.7 (9.1, 12.5)	15.2 (13.5, 17.2)	6.1 (4.8, 7.8)
**Maternal Age**						
15–19	1280	8.4 (7.6, 9.3)	59.0 (53.6, 64.3)	15.7 (11.5, 21.2)	19.1 (15.1, 23.8)	6.2 (4.6,8.1)
20–24	3784	25.5 (24.2, 26.8)	64.2 (61.4, 66.9)	12.7 (10.8, 14.9)	16.4 (14.5, 18.4)	6.7 (5.5,8.2)
25–29	4253	29.2 (27.8, 30.6)	65.5 (62.7, 68.1)	11.8 (10.1, 13.7)	15.4 (13.4, 17.5)	7.4 (5.9, 9.2)
30–34	3073	22.6 (21.3, 24.0)	69.1 (65.7, 72.3)	10.2 (8.2, 12.7)	13.7 (11.6, 16.2)	6.9 (5.1, 9.3)
35 +	1883	14.3 (13.3, 15.5)	70.7 (66.9, 74.2)	7.6 (6.0, 9.6)	16.9 (14.0, 20.3)	4.8 (3.4, 6.6)
**Education**						
< HS	3141	18.1 (16.9, 19.4)	53.6 (50.2, 57.0)	13.5 (11.2, 16.3)	23.6 (20.9, 26.5)	9.3 (7.5, 11.4)
High school/GED	3664	34.0 (32.6, 35.5)	63.8 (61.0, 66.5)	13.0 (11.2, 15.2)	16.5 (14.5, 18.6)	6.7 (5.4, 8.3)
Some college	5174	24.4 (23.1, 25.7)	67.4 (65.0, 69.7)	11.9 (10.3, 13.8)	14.1 (12.5, 15.9)	6.6 (5.5, 7.9)
College grad/ +	2294	23.5 (22.2, 24.5)	83.5 (80.1, 86.4)	5.5 (4.4, 6.8)	7.9 (5.8, 10.8)	3.1 (1.6, 5.9)
**Primary Language spoken at home**						
English	8115	50.9 (49.5, 52.4)	70.6 (68.8, 72.4)	10.0 (8.9, 11.2)	12.7 (11.5, 14.0)	6.7 (5.6, 7.9)
Spanish	3487	26.6 (25.3, 28.0)	57.7 (54.6, 60.7)	13.1 (11.0, 15.5)	22.5 (20.1, 25.0)	6.8 (5.4, 8.4)
Other	2671	22.5 (21.1, 23.9)	66.3 (62.6, 69.7)	12.6 (10.3, 15.3)	14.9 (12.3, 17.9)	6.3 (4.8, 8.3)
**Number of people living on the household income**						
≤ 2	5184	35.5 (34.0, 37.0)	67.4 (65.0, 69.7)	11.8 (10.3, 13.6)	15.4 (13.6, 17.3)	5.4 (4.4, 6.5)
3–4	6966	48.8 (47.2, 50.3)	67.1 (64.9, 69.1)	11.3 (9.9, 12.9)	14.9 (13.5, 16.5)	6.7 (5.6, 8.1)
≥ 5	2123	15.8 (14.7, 17.0)	60.8 (56.9, 64.6)	10.7 (8.6, 13.3)	19.4 (16.4, 22.7)	9.1 (7.1, 11.7)
**Insurance Coverage During Pregnancy**						
Private	3308	30.9 (29.6, 32.3)	79.9 (77.7, 82.0)	8.0 (6.7, 9.6)	9.4 (7.9, 11.2)	2.7 (2.0, 3.6)
Medi-Cal	10,051	62.0 (60.7, 63.4)	58.7 (56.8, 60.7)	13.1 (11.8, 14.6)	19.4 (18.0, 20.9)	8.8 (7.6, 10.0)
Uninsured	350	2.4 (1.9, 2.9)	51.7 (40.9, 62.3)	14.4 (8.6, 23.1)	23.2 (14.9, 34.5)	10.7 (6.2, 17.8)
Other	564	4.7 (4.2, 5.4)	81.4 (75.1, 86.4)	9.2 (5.2, 15.7)	6.8 (4.6, 9.9)	2.6 (1.2, 5.3)
**Household Income (% FPG)**						
0 – 100%	7972	54.0 (52.5, 55.4)	55.6 (53.7, 58.0)	13.4 (12.0, 15.0)	21.0 (19.4, 22.6)	9.8 (8.5, 11.2)
101 – 200%	3713	25.1 (23.8, 26.5)	70.5 (67.6, 73.3)	11.3 (9.5, 13.4)	13.8 (11.7, 16.2)	4.4 (3.4, 5.7)
201 – 300%	1503	11.8 (10.9, 12.8)	85.4 (82.1, 88.3)	7.0 (5.0, 9.7)	5.9 (4.1, 8.3)	1.7 (1.0, 2.9)
301 – 400%	1085	9.1 (8.4, 10.0)	90.5 (87.3, 93.0)	5.6 (3.7, 8.4)	3.6 (2.2, 5.8)	0.3 ( 0.1, 0.8)
**Marital Status**						
Married	6773	50.0 (48.5, 51.5)	73.2 (71.2, 75.0)	9.3 (8.2, 10.7)	13.4 (12.0, 15.0)	4.0 (3.3, 4.9)
Living together	4472	30.3 (28.9, 31.8)	59.8 (57.0, 62.5)	12.1 (10.4, 14.0)	18.8 (16.8, 21.0)	9.4 (7.9, 11.1)
Single	3028	19.7 (18.5, 20.9)	58.4 (54.8, 61.9)	15.6 (12.9, 18.8)	17.1 (14.8, 19.7)	8.9 (6.8, 11.7)
**Race/Ethnicity**						
White	4013	24.2 (23.1, 25.3)	74.7 (72.5, 76.8)	8.7 (7.5, 10.2)	10.7 (9.2, 12.3)	5.9 (4.9, 7.1)
Black	1357	6.7 (6.2, 7.2)	65.8 (60.0, 71.5)	11.3 (8.3, 15.2)	15.0 (11.6, 19.1)	7.9 (4.2, 14.3)
Latina foreign-born	4009	30.0 (28.6, 31.4)	55.1 (52.2, 58.0)	13.6 (11.5, 16.0)	23.5 (21.2, 25.9)	7.8 (6.3, 9.7)
Latina U.S.-born	3695	29.3 (27.9, 30.8)	68.3 (65.3, 14.0)	11.9 (10.0, 14.0)	13.6 (11.6, 15.9)	6.3 (5.0, 7.9)
Asian/PI	1199	9.9 (8.9, 11.0)	73.1 (68.1, 77.6)	9.9 (7.1, 13.7)	12.0 (8.8, 16.1)	5.0 (3.5, 7.2)

*The Total column sums to 100% vertically for each variable while other columns sum to 100% across the Food secure, Marginally food secure, and the All food insecure columns

**Fig. 2:**
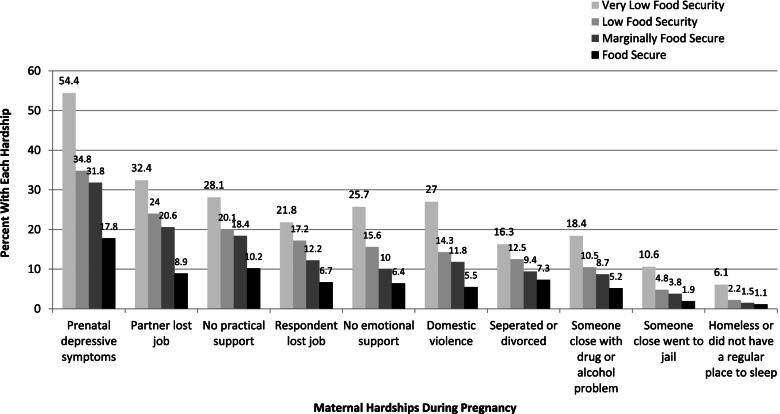
Percent of women with household incomes < 400% of Federal Poverty Guidelines in California experiencing serious hardship during pregnancy by food security status*, MIHA 2010 – 2012 (*n* = 14,274)

For each of the ten maternal hardships that were assessed, a stepwise gradient in hardship prevalence was observed by food security status (Fig. 2). The prevalence estimates for each maternal hardship were statistically significantly higher for food-insecure women compared to women who were food-secure (*p* < 0.05). The most common maternal hardship for any category of food insecurity status was prenatal depressive symptoms, with over one-half (54.4%) of women with very low food security reporting depressive symptoms during pregnancy, and one-third of women with low or moderate food security reporting those symptoms (34.8% and 31.8%, respectively). Three out of four women who reported food insecurity experienced at least one additional maternal hardship (data not shown).

The results from the adjusted multinomial logistic regression for the associations between food security status and the ten additional severe maternal hardships are shown in Table 3. The relative risk ratio for each maternal hardship on food security status, after adjusting for demographic and socioeconomic factors, was statistically significant for all but one hardship; having someone close who had an alcohol or drug problem. Having a husband or partner lose a job, experiencing depressive symptoms, having no practical support, and experiencing intimate partner violence were statistically and significantly associated with food insecurity consistently across all levels, with the point estimate much higher among the very low food secure group, after adjustment with a number of socioeconomic factors. The respondent losing her job was associated with both low and very low food security. Not having emotional support, the respondent or someone close to the respondent going to jail, and homelessness were associated only with very low food security. While elevated, the respondent or someone close to the respondent having a problem with alcohol or drugs was not significantly associated with any level of food insecurity. Our sensitivity analysis, which included women with imputed household incomes, produced consistent results (data not shown).

**Table 3:** Adjusted relative risk ratios for the associations between several maternal hardships and food security status, adjusting for socioeconomic characteristics among postpartum women with incomes ≤ 400% FPG in California, 2010 – 2012 (*n* = 14,274)

	**Marginally Food Secure**			**Low Food Security**			**Very Low Food Security**		
	Adjusted RRR	95% CI		Adjusted RRR	95% CI		Adjusted RRR	95% CI	
**Maternal Hardships**									
Separated or divorced	0.67^a^	0.47	0.95	1.08	0.79	1.48	0.85	0.56	1.29
Husband / Partner lost job	2.12^c^	1.57	2.87	2.30 ^c^	1.82	2.91	2.74 ^c^	2.00	3.76
Depressive Symptoms	1.75^c^	1.36	2.23	1.7 2 ^c^	1.40	2.11	3.26 ^c^	2.46	4.32
No Practical Support	1.72^c^	1.25	2.37	1.38 ^a^	1.02	1.87	1.81 ^b^	1.22	2.70
Inter-partner Violence	1.61^a^	1.09	2.38	1.73 ^c^	1.31	2.27	2.65 ^c^	1.86	3.79
Respondent lost job	1.33	0.94	1.87	1.80 ^c^	1.37	2.38	1.90 ^c^	1.29	2.82
No Emotional Support	0.81	0.55	1.19	1.29	0.94	1.76	1.64 ^a^	1.07	2.52
Respondent/Someone close went to jail	1.23	0.76	2.00	1.33	0.88	2.00	2.01 ^b^	1.29	3.12
Homeless or did not have a regular place to sleep	0.85	0.40	1.81	0.96	0.51	1.82	1.98 ^a^	1.08	3.63
Someone close had a problem with drugs or alcohol	1.21	0.86	1.72	1.31	0.96	1.79	1.23	0.82	1.84
**Maternal Age**									
30–34 vs. 35 +	1.42	0.99	2.03	0.88	0.65	1.21	1.46	0.88	2.42
25–29 vs. 35 +	1.56^b^	1.12	2.17	0.93	0.69	1.25	1.44	0.91	2.30
20–24 vs. 35 +	1.43	1.00	2.06	0.89	0.65	1.22	1.04	0.64	1.69
15–19 vs. 35 +	1.58	0.97	2.57	0.99	0.66	1.49	0.79	0.46	1.36
**Education**									
Some college vs. ≥ College	2.01^c^	1.42	2.85	1.40	0.96	2.03	1.34	0.75	2.38
High school/GED vs. ≥ College	1.87^b^	1.27	2.75	1.24	0.83	1.84	1.17	0.63	2.17
≤ High School vs. ≥ College	1.96^c^	1.29	2.97	1.61 ^a^	1.07	2.42	1.36	0.73	2.54
**Language spoken at home**									
Spanish vs. English	0.91	0.61	1.36	0.80	0.57	1.13	0.42 ^b^	0.24	0.74
Other vs. English	1.13	0.84	1.52	0.88	0.64	1.19	0.77	0.50	1.19
**Number of people living off of the household income**									
3–4 vs. ≤ 2	1.00	0.79	1.26	0.92	0.75	1.13	1.39 ^a^	1.02	1.90
≥ 5 vs. ≤ 2	0.92	0.66	1.27	1.08	0.81	1.43	1.72 ^b^	1.16	2.53
**Insurance Coverage**									
Private vs. Medi-Cal	0.94	0.69	1.27	0.97	0.74	1.28	0.81	0.51	1.30
Uninsured vs. Medi-Cal	1.35	0.77	2.37	1.32	0.71	2.46	1.33	0.71	2.49
Other vs. Medi-Cal	0.90	0.45	1.79	0.56 ^a^	0.35	0.88	0.62	0.27	1.41
**Household Income (% of poverty Guidelines)**									
201 – 300% vs. 301 – 400%	1.13	0.65	1.96	1.61	0.85	3.06	5.04 ^b^	1.83	13.89
101–200% vs. 301 – 400%	1.58	0.96	2.60	3.58 ^c^	1.98	6.46	11.21 ^c^	4.23	29.70
0 – 100% vs. 301 – 400%	1.81^a^	1.07	3.05	5.18 ^c^	2.83	9.48	21.51 ^c^	7.86	58.84
**Marital Status**									
Living with someone vs. Married	1.10	0.84	1.43	1.05	0.85	1.29	1.74 ^b^	1.25	2.41
Single vs. Married	1.53^b^	1.11	2.10	0.86	0.66	1.13	1.48	0.96	2.29
**Race/Ethnicity**									
Latina U.S.-born vs. White	0.99	0.74	1.33	0.93	0.71	1.22	0.72	0.50	1.02
Latina foreign-born vs. White	1.48	0.99	2.20	1.81 ^c^	1.27	2.60	1.40	0.81	2.40
Black vs. White	0.91	0.61	1.36	0.94	0.64	1.38	0.62	0.37	1.05
Asian/PI vs. White	1.22	0.81	1.83	1.23	0.83	1.83	1.02	0.60	1.74
**Year**									
2011 vs. 2010	1.00	0.78	1.29	1.06	0.85	1.32	1.02	0.75	1.39
2012 v. 2010	0.91	0.71	1.16	0.98	0.78	1.23	0.83	0.61	1.13

Statistically significantly differs from food secure at p-values of: a = *p* < 0.05, b = *p* < 0.01, c = *p* < 0.001

A final multinomial logistic regression model was fit to estimate the association between the total number of severe maternal hardships a woman had (0–10) and food security status, adjusting for demographic and socioeconomic factors. Each additional severe maternal hardship a woman experienced during pregnancy was associated with a 36% greater risk of reporting marginal food security (RRR 1.36, 95% CI: 1.27, 1.47), 54% for low food security (RRR 1.54, 95% CI: 1.44, 1.64), and 99% for very low food security (RRR 1.99, 95% CI: 1.83, 2.15) (data not shown).

## Discussion

Food insecurity was very common in this large sample of California women representative of household with incomes below 400% of the federal poverty guidelines. Almost a quarter of women reported either being low or very low food secure during pregnancy; with an additional 11.5% reporting being marginally food secure. During this time period (2010–2012), the prevalence of food insecurity in the US was 14.7% and in California 15.6% [25]. Furthermore, this study found food insecurity was seldom the only severe maternal hardship faced by pregnant women. As expected, prevalence of food insecurity during pregnancy was higher among low-income women; however, food insecurity was reported among women with household incomes as high as 300–400% of the federal poverty guidelines. This may suggest that federal, state and local assistance programs should relax strict income criteria for program participation. During pregnancy, many families experience dynamic employment and financial changes [2]. Women from low-income households as well as those who were not married, lived in larger families, and reported additional maternal hardships were associated with greater risk for food insecurity.

Similar to those who reported low or very low food security, women who reported marginal food security during pregnancy were less educated, had lower-income, were not married, and more likely to report maternal hardships compared to their food secure counterparts. These results support the notion that women facing marginal food security are not only distinct from women who are fully food secure but have vulnerabilities comparable to those experienced by women reporting food insecurity [3, 5, 26], and therefore marginal food security should be assessed, monitored, and addressed in addition to food insecurity [24], especially during pregnancy.

While it is unsurprising that food insecurity is associated with an array of maternal hardships, the prevalence and magnitude of the co-occurrences has not been previously measured. The vast majority of women who experienced marginal, low or very low food security reported one or more additional maternal hardships. Most notable were depressive symptoms, job loss, lack of practical support, and intimate partner violence as these were among the most prevalent and were associated with all levels of food insecurity. At greatest risk were the over seven percent of women who reported very low food security during pregnancy. This group had the highest prevalence of all maternal hardships reported. These findings suggest that pregnant women experiencing food insecurity not only face a threat to their nutritional status but also to their overall well-being and the health of their fetus. The strong and consistent association between levels of food security and number of maternal hardships, independent of demographic and socioeconomic factors, may suggest that food security status is a reflection of major adverse life events and could be used for screening for additional social needs.

A causal relationship between food security status and severe maternal hardships was not the focus of this analysis and is difficult to determine; causal direction cannot be inferred. However, the strong association between food security status and severe maternal hardships suggest that both must be addressed during prenatal care, nutritional services, and other social services. An excellent example of organized screening efforts are guidelines from the American Academy of Pediatrics (AAP) [27] and the American College of Obstetrics and Gynecology (ACOG) [28] that promote screening and referrals for social determinants of health, food security, maternal depression, maternal hardships, and to bolster emotional, physical and social support, although consistency of screening implementation is not known. The California Department of Public Health also recommends that providers screen for these social issues and food security during pregnancy within the comprehensive *Initial and Trimester Assessment and Care Plan* Program [29] through the Comprehensive Perinatal Services Program. The current AAP policy statement encourages pediatricians to promote food security for all families with young children in pediatric settings by screening for food insecurity and other social needs at routine health maintenance visits as well as to advocate at the local, state and federal level for policies and programs that support acquisition of nutritious foods for all families [30]. These guidelines for screening must be instituted as a standard of care within the prenatal care setting to ensure social needs of pregnant women are being met. Until we have an effective preventive approach, we must put in place and bolster routine screening and referral services—not just at public hospitals/clinics but at most prenatal care sites. Furthermore, nutrition programs like the Women, Infant and Children Supplemental Nutrition Program (WIC) should have the resources to screen and provide referrals on a wide range of maternal hardships. Social policies could address the attendant stress-related outcomes and mental and somatic health consequences that result from these severe maternal hardships by ensuring enrollment in safety net programs and designing new interventions, as done in other industrialized countries, to shield pregnant women from falling into or worsening their poverty condition [31]. Points of interventions and referral must be identified to assist with communication among programs.

Our cross-sectional analysis may have been subject to selection bias, as the severely food insecure women and the women who faced the most hardships may have consistently chosen not to participate in the survey. Our analysis would subsequently underestimate the prevalence of food insecurity in pregnancy and potentially its co-occurrence with additional hardships. Additionally, our data is roughly eight years old and examines only Californian women which reduces its generalizability. However, state representative data on pregnant women and hardships is difficult to procure, and has not been subsequently analyzed. Additionally, while there have been changes in the California economy, it is unclear if the relationships between food insecurity and hardships would change drastically over time. A key strength of this study, is that it is the first to our knowledge that measured maternal hardships in pregnancy in a representative sample of low- and lower-income pregnant women and examined the co-occurrence of hardships with food security status. While this paper did not seek to nor identify causal relationships between food security status and these various hardships, by measuring and highlighting the co-occurrence of food insecurity and hardships, this paper contextualizes hardships faced by low- and lower-income pregnant women in California for programs that aim to help them.

### Public health implications

Targeted screening for severe maternal hardships is necessary to identify needed services and provide referrals, especially among socioeconomically disadvantaged maternity populations. An example of the importance of real time screening is the Prenatal Risk Overview, a screening mechanism created by the Minneapolis Health Department that was used to assess the prevalence of psychological risk during pregnancy among low-income women seeking prenatal service in four community clinics [32]. Use of this screener between 2005 and 2007 identified high frequency of psychological risk among pregnant women: 75% had a lack of social support, 48% housing instability, 32% food insecurity, 25% drug use, 23% smoking and alcohol use, 18% depression, 9% physical/sexual abuse and 7% partner violence. Sixty percent were classified as high risk in one or more domains. Research has found that among African-Americans, each additional negative life event has been associated with lower gestational age, and higher levels of event distress were associated with lower birth weight [13]. Therefore, the number of hardships faced is itself a predictor of adverse outcomes, over and above the nature of any particular hardship. More work is needed to help pregnant women address each of the several maternal hardships that they may face during pregnancy.

Food insecurity is a very common adverse pregnancy condition, and has been associated with financial hardship, stress and mental health, as well as poor nutrition. Food insecurity has persisted at the same levels for the last decade [4, 17], suggesting that food insecurity must be addressed with multifactorial strategies [3]. Federal programs that address food insecurity and well-being are more critical than ever when one in every three pregnant women of moderate- or low-income face food insecurity. Because food insecurity is often not an isolated problem, programs that provide services to pregnant women need to not only screen for food insecurity but other severe hardships as well, to bolster referrals and help inform future interventions. Strategies must address social inequities and eliminating root causes of health disparities. Interventions are needed that go beyond acute situations and change the cycle of poverty, food insecurity, hardship and health outcomes for future generations.

## Data Availability

The Maternal Infant Health Assessment is an annual state-wide survey of women who had a recent live birth. The data are collected and archived through the California Department of Public Health. MIHA Data Snapshots are available here: https://www.cdph.ca.gov/Programs/CFH/DMCAH/MIHA/Pages/Data-and-Reports.aspx The data used for this project were analyzed under contract with the California Department of Public Health and are currently not available to researchers outside of the California Department of Public Health.

## References

[1] Gamba R, Leung CW, Guendelman S, Lahiff M, Laraia BA (2016). Household Food Insecurity Is Not Associated with Overall Diet Quality Among Pregnant Women in NHANES 1999–2008. Matern Child Health J.

[2] Park CY, Eicher-Miller HA (2014). Iron deficiency is associated with food insecurity in pregnant females in the United States: National Health and Nutrition Examination Survey 1999–2010. J Acad Nutr Diet.

[3] Laraia BA, Siega-Riz AM, Gundersen C, Dole N (2006). Psychosocial factors and socioeconomic indicators are associated with household food insecurity among pregnant women. J Nutr.

[4] Braveman P, Marchi K, Egerter S (2010). Poverty, near-poverty, and hardship around the time of pregnancy. Matern Child Health J.

[5] Laraia BA, Siega-Riz AM, Gundersen C (2010). Household food insecurity is associated with self-reported pregravid weight status, gestational weight gain, and pregnancy complications. J Am Diet Assoc.

[6] Laraia B, Epel E, Siega-Riz AM (2013). Food insecurity with past experience of restrained eating is a recipe for increased gestational weight gain. Appetite.

[7] Borders AE, Grobman WA, Amsden LB, Holl JL (2007). Chronic stress and low birth weight neonates in a low-income population of women. Obstet Gynecol.

[8] Carmichael SL, Yang W, Herring A, Abrams B, Shaw GM (2007). Maternal food insecurity is associated with increased risk of certain birth defects. J Nutr.

[9] Roseboom TJ, Painter RC, van Abeelen AF, Veenendaal MV, de Rooij SR (2011). Hungry in the womb: what are the consequences?. Lessons from the Dutch famine Maturitas.

[10] Project TLCM. Life Course Indicator: Household Food Insecurity. Washington, D.C.: Association of Mternal & Child Health Programs;2013–2014.

[11] Laraia BA (2013). Food Insecurity and Chronic Disease. Adv Nutr.

[12] Laraia B, Vinikoor-Imler LC, Siega-Riz AM (2015). Food insecurity during pregnancy leads to stress, disordered eating, and greater postpartum weight among overweight women. Obesity (Silver Spring).

[13] Dominguez TP, Schetter CD, Mancuso R, Rini CM, Hobel C (2005). Stress in African American pregnancies: testing the roles of various stress concepts in prediction of birth outcomes. Ann Behav Med.

[14] Entringer S, Kumsta R, Hellhammer DH, Wadhwa PD, Wust S (2009). Prenatal exposure to maternal psychosocial stress and HPA axis regulation in young adults. Horm Behav.

[15] Entringer S, Epel ES, Kumsta R (2011). Stress exposure in intrauterine life is associated with shorter telomere length in young adulthood. PNAS.

[16] Entringer S, Wust S, Kumsta R (2008). Prenatal psychosocial stress exposure is associated with insulin resistance in young adults. AJOG.

[17] 2012 MIHA County Report: A Summary Report of County Snapshots and Geographic Comparisons from the Maternal and Infant Health Assessment Survey. Sacramento: California Department of Public Health, Maternal, Child and Adolescent Health Program; 2014. https://www.cdph.ca.gov/Programs/CFH/DMCAH/MIHA/CDPH%20Document%20Library/MIHA-AnnualReport-2012-County.pdf . Accessed November 1, 2021.

[18] Project TLCM (2013). Life Course Indicator: Stress During Pregnancy.

[19] Dumont DM, Wildeman C, Lee H, Gjelsvik A, Valera P, Clarke JG (2014). Incarceration, maternal hardship, and perinatal health behaviors. Matern Child Health J.

[20] Heflin CH, Butler JS (2013). Why Do Women Enter and Exit From Material Hardship?. J Fam Issues.

[21] Office of the Assistant Secretary for Planning and Evaluation. 2011 HHS Poverty Guidelines. In: Services US DoHH, ed. FEDERAL POVERTY MEASURE. Vol 76. Washington, D.C.: Federal Register 2011:3637-3638.

[22] US DoHaUD. Estimated Median Family Incomes for Fiscal Year 2011. US Department of Housing and Urban Development. Washington, D.C. 2011. https://www.huduser.gov/portal/datasets/il/il11/medians2011_sig.pdf. Accessed November 3, 2021.

[23] Blumberg SJ, Bialostosky K, Hamilton WL, Briefel RR (1999). The effectiveness of a short form of the Household Food Security Scale. Am J Public Health.

[24] Coleman-Jensen A (2010). US Food Insecurity Status: Toward a Refined Definition. Soc Indic Res.

[25] Coleman-Jensen A, Nord M, Singh A (2013). Household Food Security in the United States in 2012, ERR-155.

[26] Cook JT, Black M, Chilton M, et al. Are food insecurity’s health impacts underestimated in the U.S. population? Marginal food security also predicts adverse health outcomes in young U.S. children and mothers. Adv Nutr. 2013;4(1):51–61. 10.3945/an.112.003228 PMC3648739 23319123

[27] American Academy of Pediatrics (AAP). Screening Technical Assistance & Resource Center. Child Development, Maternal Depression, Social Determinants of Health 2021. Available at: https://www.aap.org/en/patient-care/screening-technical-assistance-and-resource-center/. Accessed 2 Nov 2021.

[28] Committee on Health Care for Underserved Women (2018). ACOG Committee Opinion No. 729: Importance of Social Determinants of Health and Cultural Awareness in the Delivery of Reproductive Health Care. Obstet Gynecol.

[29] Program CCPS. CPSP Initial and Trimester sample combined assessment and care plan 5/2014. 2014; https://www.cdph.ca.gov/Programs/CFH/DMCAH/CPSP/CDPH%20Document%20Library/CPSP-CombinedInitialandTrimesterAssessmentandCarePlan.pdf. Accessed July 13, 2021, 2021.

[30] Council On Community P, Committee On N (2015). Promoting Food Security for All Children. Pediatrics.

[31] Larson CP (2007). Poverty during pregnancy: Its effects on child health outcomes. Paediatr Child Health.

[32] Harrison PA, Sidebottom AC (2008). Systematic prenatal screening for psychosocial risks. JHCPU.

